# The relationship of severity of symptoms of depression, anxiety, and stress with sleep quality in earthquake survivors in Kermanshah

**DOI:** 10.5249/jivr.v11i2.1203

**Published:** 2019-07

**Authors:** Amir Bavafa, Habibolah Khazaie, Behnam Khaledi-Paveh, Leeba Rezaie

**Affiliations:** ^*a*^Department of Clinical Psychology, Faculty of Medicine, Kermanshah University of Medical Sciences, Kermanshah, Iran.; ^*b*^Sleep Disorders Research Center, Kermanshah University of Medical Sciences, Kermanshah, Iran.; ^*c*^Department of Psychiatric Nursing, School of Nursing and Midwifery, Kermanshah University of Medical Sciences, Kermanshah, Iran.

**Keywords:** Anxiety, Depression, Earthquake, Sleep, Stress

## Abstract

**Background:**

Earthquake is one of the most common natural disasters. A 7.3" Richter earthquake happened at 5km from the town of Ezgeleh in Kermanshah province in 2017, which caused several physical and mental injuries. The present study was conducted to investigate the sleep quality and mental health difficulties of those affected by earthquake and predict sleep quality according to severity of symptoms of depression, anxiety, and stress in the township of Sarpol-e Zahab, which suffered the most damage.

**Methods:**

A total of 999 earthquake survivors living in temporary tents and camps were assessed in terms of sleep quality and pattern using Pittsburgh Sleep Quality Index, and severity of psychological symptoms using Depression, Anxiety, and Stress scale 10 days after the disaster.

**Results:**

According to the results, poor sleep quality was experienced by 20.61% of survivors, severe stress by 60.5%, and severe depression by 41.5%, and moderate anxiety by 74%. The subjective quality, efficiency, daily dysfunction, use of hypnotics, and total sleep quality had a positive and significant relation with severity of experienced depression, anxiety, and stress. Sleep latency had a positive and significant relation only with stress, and sleep disturbance with depression and stress.

**Conclusions:**

Severity of depression, anxiety, and stress can predict changes in total sleep quality of those affected by earthquake. Stress can be considered as the sole predictor of total sleep quality and the only factor that can explain components of sleep quality. The implications of the present study are debatable.

## Introduction

In recent years, numerous natural disasters have happened throughout the world, of which, earthquake is one. Compared to other natural disasters, earthquakes are uncontrollable, cause damage faster and without warning, and affect greater number of people.^1^ Developing countries experience more severe consequences compared to developed countries, as such, health problems can be referred to. This issue can be partly due to the limited access to resources in general, and resources for treatment of psychological and physical problems in particular. However, a relatively low number of post-earthquake research studies have been conducted in developing countries due to low levels of funding and public interest.^2^ A study reviewed 116 studies conducted on psychological health consequences of natural disasters, of which, only 40 had been conducted in developing countries. ^3^ Hence, the need is felt for further studies on long and short-term consequences of severe earthquakes in these countries. 

On November 12th 2017, a 7.3 Richter earthquake occurred at 5km from the town of Ezgeleh in Kermanshah province western part of Iran. Local data reveals that more than 600 people died, and 79000 were injured or became homeless, respectively. The individuals of Sarpol-e Zahab county (in Kermanshah province) suffered the most (http://www.lmo.ir).

Several studies have reported mental health difficulties following earthquakes,^4-10^ but there are few reliable studies on this earthquake. ^11-13^ Previous studies have mostly focused on the prevalence of posttraumatic stress, depression, anxiety, such like^6^ and^14,15^ less on sleep problems and concurrent psychological symptoms in earthquake survivors. In a study, the sleep problems of the survivors of the great east Japan earthquake were studied and the findings suggest that the frequency of more nocturnal sleep problems causes more problems in treating insomnia. This study also showed that serial nocturnal nightmares in people nearer to the disaster site occur more abundantly.^16^ A study by Nobakht & Dale in 2019 found that sleep disorders and nightmares could be considered as a mediator in the relationship between trauma and dissociation.^12^ This showed that sleep problems can persist long after the event and cause problems in returning to daily life and responding to treatment. In several studies,^17-19^ post traumatic stress is considered as major component of sleep disturbances. And thus it is important to investigate sleep problems and damages they cause, since they are related to daytime turmoil and problems. ^20^

Since sleep and its related problems are one of the main sub-criteria of posttraumatic stress disorder, especially in relation to intrusion and arousal, investigating them can further help comprehensive understanding of this disorder, and since natural disasters as harmful events complete the first criterion for posttraumatic stress disorder, it was decided to investigate survivors of an earthquake as a natural disaster, to better clarify dimensions of this disorder. According to DSM-IV-TR, diagnostic criteria for posttraumatic stress include re-experience, avoidance, numbness, and arousal, and also presence of a harmful event resulting in a real risk or threat to death. These criteria have been modified in the fifth edition (DSM-5) to intrusion, avoidance, arousal, and negative alterations in cognition.^21^

The present study was conducted to investigate sleep quality and mental health difficulties of those affected by earthquake and predict sleep quality based on depression, anxiety and stress among survivors of Kermanshah earthquake. 

## Methods 

The statistical society in the present descriptive study consisted of rural and urban survivors of the 7.3 Richter earthquake in Kermanshah province (western Iran). Sampling was purposeful and available. The process of data collection began 10 days after the earthquake and lasted for two weeks. Questionnaires were initially disturbuted among 1200 residents of Sarpol-e Zahab county (epicenter of earthquake), and ultimately 999 people (437 men, 562 women) completed the questionnaires. questionnaires The remaining questionnaires were omitted from the study due to their lack of completeness (201). Data were analyzed in SPSS-IBM22. The study inclusion criteria were: minimum 15 years of age, ability to read and write, desire to participate in research and completion of questionnaires. Participants had to be indigenous to the region, with at least 5 years of residency. Questionnaire was completed in the presence of researchers, and necessary guidance was provided. Participation in the present study was on voluntary basis and with complete consent of participants. The present study was registered at the Sleep Disorders Research Center of Kermanshah University of Medical Sciences, and approved by the university ethics committee. 

Pittsburgh Sleep Quality Index (PSQI) was used to assess sleep quality and pattern. This questionnaire assesses good and bad sleep over the last month using 19 items in seven components. Each item scores between zero and three points, with the maximum score for each component of three points. The sum of scores of these seven components makes up the total score, which ranges from 0 to 21 points. Higher scores indicate poorer sleep quality. Scores higher than five suggest unfavorable sleep quality.^22^ Reliability of PSQI has been reported with Cronbach's alpha of 0.8, and test-retest reliability between 0.93 and 0.98. ^23^ Many studies have used PSQI and found it to be highly reliable.^22,24-26^ In Iran, Cronbach's alpha of this questionnaire is calculated by Moghadam et al., 0.77, respectively. ^25^

The severity of depression, anxiety and stress was assessed using DASS-21, ^27^ which is the short form of this scale and contains three seven-item parts. Despair, low self-esteem, and low positive emotion are measured by the depression part (DASS-D), autonomous arousal, excessive physiological arousal, and subjecting feeling of fear by anxiety part (DASS-A), and stress due to frequent symptoms of tension, restlessness, and negative emotion through the tress part (DASS-S). ^28^ This scale can be used in screening for general psychological turmoil as well as the severity of main symptoms of depression, anxiety and stress.^29^ This scale has been designed according to Likert Scale, with options: "Never, a little, medium and high". Each item scores between zero and three points. ^27^ This scale has shown a high reliability from 0.9 to 0.97 in different populations.^29,30^ Validity and reliability of this scale have also been assessed in Iran. ^31^

Data were analyzed by SPSS-IBM22. Independent t-test, and variance analysis were performed to determine the difference between levels of demographic characteristics. Pearson correlation was used to determine the relationship between the target components. Canonical correlation indices were used to determine the most important predictors of sleep quality based on depression, anxiety and stress. Multiple regression was conducted to predict sleep quality based on depression, anxiety and stress. 

## Results

A total of 999 earthquake survivors were assessed, of whom, 437 (43.7%) were men. Participants' mean age was 34.44 years. Mean age was 35.95 years in men and 33.04 years in women. Most participants were married (60.5%). Table 1 shows demographic characteristics and total sleep quality of subjects, with a significant difference in terms of total sleep quality. It can be seen that total sleep quality is affected by age range (P<0.001), gender (P=0.002), and marital status (P=0.001).

**Table 1:** Participants' demographic characteristics and total sleep quality.

Variable	Category	n (%)	Sleep quality	
			Mean + SD	P
**Age**	15-30	424 (49.82)	4.11+2.96	
	31-45	232 (27.23)	4.50+3.41	**<0.001**
	46-60	149 (17.48)	5.02+3.91	
	60 to Up	46 (5.47)	7.13+4.69	
**Sex**	Male	383 (44.63)	4.15+ 3.11	**0.002**
	Female	475 (55.37)	4.87 + 3.65	
**Marital status**	Bachelor	300 (35.21)	3.98 + 2.67	
	Married	516 (60.56)	4.86 + 3.81	**0.001**
	Divorced or Widow	36 (4.23)	5.22 + 2.98	
**Sleep quality**	Optimal	682 (79.39)	0.97+3.94	**<0.001**
	Poor	177 (20.61)	0.48+7.5	

Note: SD=Standard Deviation

According to Table 2 , severe stress and depression in 60.51% and 41.5% of participants respectively had the highest frequency of psychological symptoms. The present study was conducted to assess the relationship between each of the sleep quality indices and severity of depression, anxiety and stress, and also to predict total sleep quality based on depression, anxiety and stress. Correlation of these variables is shown in Table 3

**Table 2:** Frequency of severity of depression, anxiety, and stress.

Severity of symptoms	Depression N (%)	Anxiety N (%)	Stress N (%)
**Mild**	(14.8)116	(4.3)35	(14.58)135
**Moderate**	(33.7)263	(74)594	(24.91)231
**Severe**	(41.5)401	(21.7)175	(60.51)561

**Table 3:** Pearson Correlation Coefficient matrix between severity of depression, anxiety, and stress and sleep quality and its components.

Variable	Depression	Anxiety	Stress	Sleep quality
**Subjective sleep quality **	0.33**	0.36**	0.42**	0.77**
**Sleep latency **	0.00	0.01	0.08**	0.75**
**Sleep duration**	0.02	0.03	0.05	0.63**
**Sleep disturbance**	0.06*	0.05	0.13**	0.81**
**Use of sleep medication**	0.31**	0.33**	0.34**	0.73**
**Daytime dysfunction**	0.40**	0.34**	0.43**	0.78**
**Sleep efficiency**	0.23**	0.30**	0.29**	0.66**
**Total sleep quality**	0.20**	0.21**	0.28**	1
**M**	18.77	17.76	18.89	4.55
**SD**	10.15	9.95	9.60	3.43

Note: SD=Standard Deviation, M=Mean, *P<0.05, **P<0.001

Correlation table shows that subjective sleep quality, efficiency, daytime dysfunction, use of sleep medications and total sleep quality have a positive and significant correlation with depression, anxiety and stress (P=0.001). Total sleep quality also has a significant correlation with depression (0.2), anxiety (0.21), and stress (0.28) at a significant level of 0.001. Total sleep quality has a high and significant correlation with other components of sleep quality (Table 3).

Canonical correlation indices in Table 4 show that among predictor variables, stress has the highest relation with the first combined or fundamental variable resulting from dependent variables (Components of sleep quality). Anxiety has the lowest role. Wilk's Lambda value of 0.57 indicates significant differences between depression, anxiety, and stress in terms of sleep quality components (P<0.001). A total of 43% of variance of dependent variables (sleep quality components) is predicted by anxiety, depression, and stress.

**Table 4:** Standard coefficients, structural coefficients and other Canonical Correlation indices for assessing the relationship between sleep quality components and stress, anxiety and depression.

Variables	Coefficients		
	Standard coefficients	Structural coefficients	Common variance
**Depression**	0.12	0.90	Wilks’ Lambda= 0.57
**Anxiety**	0.11	0.89	R2= 0.43
**Stress**	0.80	0.99	F= 28.14
****			<0.001

The results presented in Table 5 show that independent variables altogether can predict 15% of changes in total sleep quality. Depression with β=-0.23 and P<0.05, and stress with β=0.57 and P<0.001) individually can predict sleep quality, but anxiety with β=0.04 and P>0.33 is unable to predict sleep quality.

**Table 5:** Multiple regression analysis of sleep quality based on depression, anxiety, and stress.

Dependent Variable	Predictor Variable	B(unstandardized)	ß (standardized)	T	Sig.	R2	P
**Sleep Quality**	Depression	-0.16	-0.23	2.09	0.04		
	Anxiety	0.03	0.04	0.97	0.33	0.15	**0.001**
	Stress	0.43	0.57	5.24	0.001		

## Discussion

Severe trauma leads to the development of posttraumatic stress disorder symptoms and comorbidity of anxiety and depression.^32^ The present study provided unique evidence of sleep quality and its components, and also severity of depression, anxiety and stress experienced by survivors of Kermanshah earthquake living temporary in tents in Sarpol-e Zahab region.

Significant differences in sleep quality were observed between different age groups (P<0.001), so that mean sleep quality reduced with higher PSQI score and aging. Reduced sleep quality in older age has also been shown in several other studies.^33,34^ Reducing melatonin hormone,^35^ brain cell decay, ^36^ neurological experienced problems, ^37^ taking medications^38^ and other reasons can affect the quality of sleep in older people.

Living alone can be regarded as a risk factor for psychological disorders and response to their treatments.^21^ Participants were mostly married (60.5%), but had poorer sleep quality compared to single people. However, many studies have found different results in this respect.^39,40^ It has been shown in these studies that married people have better sleep quality than others. As an explanation, the study subjects were survivors of earthquakes that had experienced stress in difficult conditions. Considering cultural issues, married couples probably had more intimate relationships, and their problems were mostly physical, and especially their sleep. Studies have shown that married people are more likely to use mental health interventions.^41^

The present study showed that severity of stress, depression and anxiety were positively and significantly related to subjective sleep quality, efficiency, daytime dysfunctions, use of sleep medications, and total sleep quality. Sleep latency had a positive and significant relationship only with stress, and sleep disturbances with depression and stress. Similar results were shown in a study conducted on survivors of Wenchuan-China earthquake two years after the event, ^8^ except that in the present study, in addition to subjective sleep quality, efficiency, and total sleep quality, anxiety tries to extend its effect on sleep quality indices. Moreover, in the above study, severity of depression and anxiety was the lowest (1.55% and 3.16% respectively), and had a positive and significant relationship with duration of sleep.^8^

These results contradict with those of the present study showing that compared to moderate and mild depression, severe depression had the highest frequency among survivors (41.5%), and duration of sleep had no significant relation with any of the psychological symptoms. One of the factors that can explain the difference in results is the shorter interval between timing of study and the time of earthquake. This is exactly the time interval that diagnostic criteria for depression and anxiety had not yet been completed. Of course lack of medical interventions in this period can also be effective. Moreover, most participants in this study were women (55.37%), indicating higher prevalence of depression among women compared to men.^21^ On the other hand, reference can also be made to the greater internalization of events and life problems among Iranians, which can explain the severity of depression symptoms a short period after the earthquake. The use of advertising in Jiang et al. study ^8^ to attract participants with poor sleep quality could be another reason. These results can have clinical implications, and draw greater attention to sleep disturbances that can affect psychological symptoms and disorders.

In the present study, 21.1% of participants met poor sleep quality criteria, which was lowest compared to other studies conducted on earthquake survivors. In a study conducted on survivors of Philippine's Haiyan Typhoon Earthquake, poor sleep quality was 55.8%.^42^ A cohort study conducted on 1573 adolescent survivors of Wanchuan-China earthquake, showed the prevalence of poor sleep quality of 22.6%. But, this was 28.70% and 30.18% 18 and 30 months respectively after the earthquake.^43^ The reasons for differences between previous and the present studies could be: 1) the difference in PSQI cut-off point, which was PSQI≥5 in the above study and PSQI>5 in the present study; 2) Data collection has been done with a longer interval than the present study; 3) Cultural differences, and also participants; mean age and sampling method; and 4) non-assessment of comorbid psychological disorders. Not many studies have been conducted on poor sleep quality in earthquake survivors, but several studies have been conducted on sleep problems. ^8,14^

## Conclusion

As the main result of multiple regression and also canonical correlation analyses, stress was proposed as the only predictor of total sleep quality and the factor that can explain components of sleep quality. From reasons explaining this result, it can be noted that the data collection distance from the occurrence of the event (10 to 24 days) was very short. In fact, depression and anxiety had a small role in explaining total sleep quality and its components a short time after the earthquake in Kermanshah. Levels of sleep quality components change (depending on their relations) with the severity of depression, anxiety and stress. Since survivors were still in the acute phase of stress, severity of stress can be said to have a greater role in explaining sleep problems compared to depression and anxiety. A study conducted by Gotto & Wilson showed that the Asian survivors of earthquake were more inclined to show their stress with physical symptoms,^44^ and this may have interfered with their sleep. The results of this study can help therapists in choosing the right ones for those who are still in shock and severe traumatic stress and experience severe problems with their sleep quality.

**Limitations**

There were several limitations in the present study: first, survivors' previous psychological state was not assessed. Psychological distress among earthquake survivors along with other problems can be regarded as a serious health problem for people living in such difficult conditions.^45^ Second, this study was conducted ten days after the earthquake. A number of families still lived in temporary tents and sites, and this made collection of data difficult. This might have reduces the representativeness of the sample and therefore the generalizability of the findings.

**Acknowledgments**

We thank all participants and helpers in conducting this project, especially the Sleep Disorders Research Center (SDRC) of Kermanshah University of Medical Sciences for their support and funding. 
